# Whole blood selenium concentrations in four free-ranging mammal species from central Scandinavia

**DOI:** 10.1186/s13028-025-00837-2

**Published:** 2025-11-26

**Authors:** Marianne Lian, Lucile Morcelet, Ilona Marmouget-Joyau, Boris Fuchs, Alexandra Thiel, Anne Randi Græsli, Alina L. Evans, Ilia Rodushkin, Kristoffer Nordli, Fredrik Stenbacka, Aimee Tallian, Wiebke Neumann, Jon M. Arnemo

**Affiliations:** 1 Department of Veterinary Medicine, University of Alaska Fairbanks, 2141 Koyukuk Drive, Fairbanks, AK 99775 USA; 2 Department of Forestry and Wildlife Management, University of Inland Norway, Anne Evenstads Vei 80, 2480 Koppang, Norway; 3 OSU Pytheas Institute, Aix-Marseille Université, 38 rue Frédéric Juliot Curie, 13013 Marseille, France; 4 Division of Geosciences, Luleå University of Technology, Laboratorievägen 14, 971 87 Luleå, Sweden; 5 ALS Scandinavia AB, Aurorum 10, 977 75 Luleå, Sweden; 6 Department of Wildlife, Fish and Environmental Studies, Swedish University of Agricultural Sciences, Skogsmarksgränd, 901 83 Umeå, Sweden; 7 Norwegian Institute for Nature Research, Trondheim, Norway

**Keywords:** Brown bear, Body weight, Ecotoxicology, Moose, Nutrition, Trophic level, Wolf, Wolverine

## Abstract

**Background:**

Selenium (Se) is an essential element for mammals, with a relatively narrow safety margin between deficiency and toxicity. It is involved in the function of many vital activities and systems, including antioxidants, immune system, thyroid activity, muscle metabolism, and growth by composing different proteins and enzymes. Northern Europe is a Se deficient region, and livestock have been supplemented with mineral bolus or similar for decades to counteract Se deficiency, whereas Finland even adds Se to fertilizers to supplement soil, plants, animals and humans. Relatively few studies have investigated total Se concentrations ([TSe]) in wildlife, and here we present [TSe] measured in whole blood in moose (*Alces alces*), brown bears (*Ursus arctos*), wolves (*Canis lupus*), and wolverines (*Gulo gulo*) from Norway and Sweden.

**Results:**

[TSe] in whole blood increased with the trophic level of the species: herbivorous moose < omnivorous bears < carnivorous wolves < scavenging wolverines. Compared to established reference ranges of [TSe] in domesticated species, more than half of all moose sampled and 5% of brown bears were Se deficient. Surprisingly, 49% of bears, 42% wolves and 29% wolverines had [TSe] above recommended references range for domesticated species. In general, [TSe] significantly increased with age and body weight in all sampled species, whereas for most species, there was an additional association with region, year, and season sampled, reflecting variations in Se uptake caused by the element’s geochemical properties related to bedrock and soil availability and atmospheric precipitation.

**Conclusions:**

Further studies should focus on a wider spatial distribution for these animals and especially include more wolverines to investigate the relatively high [TSe] observed in this species. We also emphasize the importance of measuring Se in poor regions for ecotoxicology studies, since Se deficiency can exacerbate heavy metal toxicosis.

## Background

 Identified as a new substance in 1817, selenium (Se) was first recognized as a toxic element [1]. The physiological importance of this trace element was first discovered in the 1950’s, identifying Se as an essential element with a particularly narrow range between deficiency and toxic concentrations [1]. Se is a micronutrient and essential mineral for mammals, and is involved in many activities and systems, including antioxidants, immune system, thyroid, muscle metabolism, and growth by composing different proteins and enzymes [2]. The biological functions of Se arise via the primary catalytic activity of selenocysteine (Sec) in 25 proteins expressed in the human proteome. Selenoproteins are present in all vertebrates and are especially important to prevent and reverse oxidative damage in the brain [3]. Se holds the potential to protect against mercury (Hg) [4], arsenic (As) and cadmium (Cd) [5], and under certain conditions lead (Pb) [6–8] mediated toxicity by sequestering these heavy metals into biologically inactive complexes and/or through the activity of Se-dependent antioxidant systems.

For several vertebrate species, Se has one of the narrowest reference ranges of all the micronutrients. For humans, this range is 81–165 µg/L in whole blood [9], whereas concentration in whole blood below 40 µg/L is recognized as deficiency, and above 400 µg/L is recognized as toxicity [10, 11], and most mammals follow a similar narrow safety margin. This makes both Se deficiency and toxicity a matter of concern. Animals experiencing Se deficiency can develop various diseases and symptoms including cardiovascular disease [12], thyroid disease [13], impaired immune response [14], and reduced viral defense [15]. Livestock experiencing Se deficiency can be affected by “White Muscle Disease”, reduced reproduction and fertility, abortions, reduced weight gain and diarrhea [16]. In contrast, Se toxicity or selenosis, refers to the replacement of sulfur (S) by Se in different metabolic reactions causing toxicosis [11, 17]. Symptoms of selenosis include alterations in keratinous tissues (hair loss, skin lesions, changes in nail and hoof structure), diarrhea, and joint and muscle pain [11, 18].

Se is present in nature in five oxidation states: the fully oxidized selenate (Se[+ VI]), selenite (Se[+ IV]), selenides (Se[-II]) and (Se[-I]), and elemental Se (Se[0]), and these Se compounds are widespread in rocks, soils, waters and air [1]. Sedimentary rocks are the primary source of Se, and it is mainly found in sandstone, quartzite and limestone. In contrast, igneous rocks and especially granite, basalt and andesite, accounting for large areas of the Earth’s surface, contain much lower concentrations making Se-deficient environments more widespread than Se-excessive ones [1, 19]. The Se content in soils and plants in Northern Europe is generally low [19]. In Norway and Sweden, veterinarians have frequently found Se deficiencies in livestock, especially in central and eastern parts of southern Norway, resulting in Se compounds being added to animal feed mixtures the last several decades [20]. Studies measured Se in soil in Innlandet county, Norway, and reported very low Se concentrations, with 94% of soil samples containing less than 0.2 mg/kg [21]. Consequently, low Se concentrations were reported in plants such as *Salix* spp., *A. flexuosa*, *S. virgaurea*, *M. preatense*, *G. sylvaticum*, *V. myrtillus*, wild grasses, sedges and rushes, and grass from cultivated pastures, with small variation between species [22]. To avoid deficiency the daily dietary requirement of Se for cattle and deer should exceed 0.02 mg/kg [23].

In contrast to livestock, concentrations of Se in wildlife have not been thoroughly studied in these regions, with a few exceptions. Moose (*Alces alces*) are large herbivorous browsers that have been used as a sentinel for spatial variations of Se uptake by herbivores in Sweden, reported to have Se liver concentrations considered to be deficient for cattle [24]. Brown bears (*Ursus arctos*) are omnivorous hibernators [25] with seasonal diet changes. During spring and summer, they feed on carrion and predate neonatal moose, whereas in late summer and fall, forest berries (*Vaccinium ssp.* and *Epretum nigrum*) dominate their diet [26]. Ants, mostly of the genera *Formica* and *Camponotus* are an important food source during the entire active season [26]. Scandinavian wolves (*Canis lupus*) are carnivores, predominantly predating on moose [27]. Wolverines (*Gulo gulo*) are carnivores with a wide spectrum of prey species, however facultative scavenging is probably the dominant way of food intake [28, 29]. Wolverines cache food items, and their diet might periodically be dominated by bone and skin tissues [30]. Here we compare [TSe] between moose, bears, wolves and wolverines on an herbivore-omnivore-carnivore continuum. The question we wanted to address in this study was how the Se concentrations present in these species change with varying trophic positions. We aimed to explore if moose, as a strictly herbivorous species, is Se deficient and if concentrations of this element in species on the other end of the continuum and with higher trophic positions (wolves and wolverines and partly bears) are affected by the same Se poor ecosystem. Furthermore, we explored spatial differences in Se concentrations between different regions in south-central Norway and Sweden.

## Methods

### Study area

**Fig. 1:**
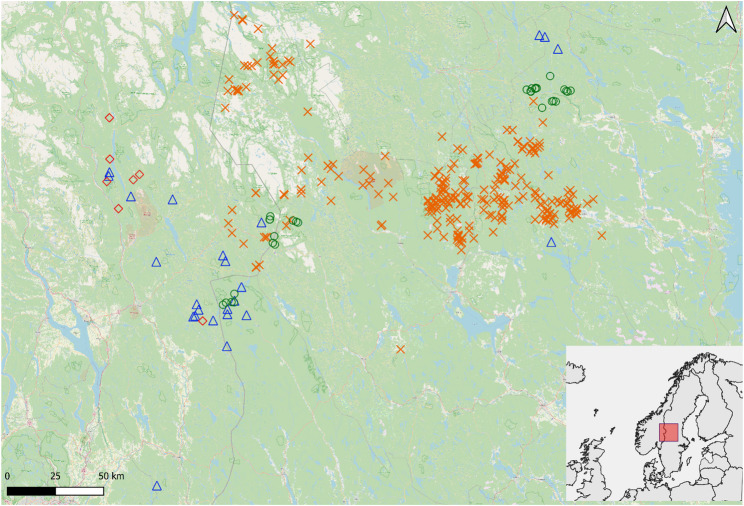
Study area. Capture positions for moose (*Alces alces*, green circle), brown bears (*Ursus arctos*, orange cross), wolves (*Canis lupus*, blue triangle), and wolverines (*Gulo gulo*, red diamond), in interior Norway and Sweden

Several long-term projects collecting and bio-banking blood samples from moose (Flisa, Inland county, Norway and Ljusdal, Gävleborg county, Sweden), brown bears (mainly Dalarna and Gävleborg counties, Sweden), wolverines (Inland county, Norway), and wolves (Inland county Norway and Värmland county, Sweden), close to the Norwegian-Swedish border contributed blood samples for this project (Fig. 1; Table 1). The area is approximately 67 699 km^2^ (~ 61 °N, 13 °E), and the region is mainly covered by cultivated and sparsely populated coniferous forest, especially Scots pine (*Pinus sylvestris*) and Norway spruce (*Picea abies*), with rolling landscape, lakes, bogs, and agricultural fields in the eastern parts. Elevation ranges between 150 m and 1400 m, where at high altitudes, the ground is covered with snow from October to mid-May, with a shorter period of snow cover at lower altitudes. Moose, bears and wolves were sampled along the country border between Norway and Sweden, whereas wolverines were sampled only in Norway (Fig. 1).

### Animal capture

We captured all animals following a detailed standardized biomedical protocol for animal anesthesia and handling techniques [31]. Moose [32], brown bears [25, 33, 34], and wolves [31] were chemically immobilized via remote injection from a helicopter, hibernating bears were darted in dens [25], while we captured wolverines using baited wooden traps and darted the animal in the trap immediately at approach [35]. All animals were sexed, and bears, wolves and wolverines were weighed using spring scales. For bears, age was either known for individuals followed from one year of age or determined by counting the cementum layer of a vestigial first premolar [36]. Similar for wolves that were captured at less than one-year-old (determined by the distal epiphyseal growth zones on radius and ulna). Age of adult wolves and wolverines is based on the time between the sampling date and the individual’s DNA first appearance in the Scandinavian carnivore monitoring database (Rovbase.no). All sampled wolves were likely DNA sampled during their first year of life, whereas for wolverines this indicates a minimum age. Moose were aged based on tooth wear. We classified age in three groups for bears and wolves: pups (wolf: ≤1 year), yearlings (bears sampled in April born the previous year), subadults (bear >2-<4 years, wolf >1-< 3), and adults (bear: ≥4 years, wolf ≥ 3 years). In certain instances, an animal was captured for the first time as either an adult or sub-adult and could therefore not be aged. Sampled wolves were either territorial adults or offspring within their natal territory. The exact location of the anesthetized animal was recorded using a hand-held GPS (Fig. 1). Capture, handling and sampling of all individuals was performed under ethical permits obtained for all different projects and is summarized in Table 1. The Scandinavian brown bear research project is a long-term research project conducting physiological and ecological studies on brown bears mainly in interior Sweden and boarder regions with Norway. The remaining projects mainly focus on ecology but additionally conduct physiological studies. All sampling for Se analyses were done opportunistically to the projects listed in Table 1, limiting our study design with regards to location and year sampled. Sampling sites were consistent across years for bears, wolves and wolverines. Moose were sampled in Flisa in 2018 and 2019, and in Ljusdal in 2020.

**Table 1:** Ethical approval document identifiers in relation to the various included research projects and host organizations

Project name	Species	Country	Hosting institution	Ethical approval document id
Scandinavian brown bear research project (SBBRP)	Brown bear (*Ursus arctos*)	Norway	Norwegian Institute for Nature Research (NINA)	FOTS ID: 19,368
		Sweden	Swedish University of Agricultural Sciences (SLU)	Dnr: 5.8.18–03376/2020
Scandinavian wolf research project	Wolf (*Canis lupus*)	Norway	University of Inland Norway (INN)	FOTS ID: 15,370
SKANDULV	Wolf (*Canis lupus*)	Sweden	Swedish University of Agricultural Sciences (SLU)	Dnr: 5.2.18–2830/16
Scandinavian forest wolverine project, Skogsjerv	Wolverine (*Gulo gulo*)	Norway	INN	FOTS ID: 19,625
Grensevilt	Moose (*Alces alces*)	Norway	INN	FOTS ID: 15,170
Viltsamverkan i brandens spår	Moose (*Alces alces*)	Sweden	SLU	Dnr: A14-15

### Blood sampling

A total of 379 blood samples (from 218 individuals) were collected from chemically immobilized animals; moose (*n* = 31, between February and March 2018–2020), brown bears (*n* = 305, between February and June 2010–2021), wolves (*n* = 26, between January and March 2014–2022) and wolverines (*n* = 17, between January and April 2017–2021). Some brown bears were re-captured throughout the study. The distribution of sample size per year is listed in Table 2. Blood samples were collected from the jugular vein for bears, moose, and wolverines, and from the cephalic vein for wolves. Whole blood samples were stored either in 4 mL evacuated EDTA tubes (EDTA, Vacuette, Greiner Bio-One International GmbH, Kremsmünster, Austria; *n* = 144) or in 6 mL evacuated heparin trace element tubes (TE; Vacuette; *n* = 204). Samples were kept cool in the field, and kept at -20 °C until permanent storage at -80 °C as soon as possible and upon arrival in the laboratory. Samples were shipped chilled to ALS Scandinavia laboratories.

**Table 2:** Sample size distributed across sampling years

Year	Moose	Brown bears	Wolves	Wolverines
2010		13		
2013		9		
2014			3	
2015			1	
2016			1	
2017		36		2
2018	5	54	3	4
2019	10	64	4	4
2020	16	72	3	1
2021		57	2	6
2022			9	
In total	31	305	26	17

A total of 379 blood samples were collected from chemically immobilized animals; moose between February and March 2018–2020, brown bears between February and June 2010–2021, wolves between January and March 2014–2022), and wolverines between January and April 2017–2021

### Se analyses

Total Se concentration ([TSe]), reported as µg/L, was determined for all whole blood (WB) samples. The samples were prepared by closed vessel micro-wave assisted nitric acid digestion [37]. Next, [TSe] were measured by sector-field inductively coupled plasma mass spectrometry (ICP-SFMS, ELEMENT XR, Thermo Scientific, Bremen, Germany) using 77Se and 78Se isotopes in high-resolution mode with combination of internal standardization and external calibration as described in detail elsewhere [38]. Quality controls included preparation blanks, parallel samples and matrix-matched test samples (Seronorm Trace Elements Whole Blood − 1 (lot 1702821), L-2 (lot 1702825) and L-3 (lott 1702826), with tabulated Se concentrations in the range 69–254 µg/L, SERO AS, Norway) prepared and analyzed in each batch of blood samples. Method quantification limit for Se, determined as 10 times the standard deviation (SD) for preparation blanks was 2 µg/L with analyte recovery in test samples within 88%-106% range. Mean in-run repeatability (assessed using relative SD (RSD) for results for parallel samples (*n* = 52) analyzed within single analytical session was 5.1% RSD. As samples for this study have been analyzed in several analytical sessions spanning few years, long term reproducibility was assessed by re-analyzing 22 randomly selected blood samples from bears, previously analyzed in four different batches as blind samples in the last analytical session. Long term reproducibility was 7.4% RSD for, thus roughly 1.5 times higher than in-run reproducibility.

### Statistical analyses

For all analyses [TSe] was used as the response variable and was tested for normality using histograms and Shapiro Wilkes test. To compare [TSe] between the different species, we used ANOVA followed by post hoc comparisons using estimated marginal means (emmeans) with Tukey’s adjustment for multiple comparisons, implemented with the “emmeans” package in R [39]. For the species-specific analyses model selection was performed using the Akaike Information Criterion (AIC) to identify the best-fitting model among a set of candidate models [40]. Model selection was performed using the dredge function from the MuMIn package [41], which systematically generates all possible sub-models of the global model and ranks them based on corrected Akaike Information Criterion (AICc). The AICc was used to correct for small sample sizes. ΔAICc < 2 was considered the best fitted model [42]. Explanatory variables are outlined in the results for each species. Model selection and comparison was implemented using R packages bbmle [43] and MuMIn. For bears, we used a linear mixed model to account for several measurements from the same animal over the years and added animal ID as a random effect to the model (R package lme4 [44]). For models selected with ΔAICc < 2 post hoc testing was performed with emmeans. A P value < 0.05 was considered significant for both post hoc testing and the ANOVA. A correlation analysis was conducted ahead of model selection using the cor() function in R (Pearson correlation). When variable pairs had a correlation >0.7, one was emitted from the model. This resulted in the following predictor variables for the different species:

- Moose: sex, estimated age and location (Flisa, Inlandet county, Norway and Ljusdal, Gävleborg county, Sweedn). Year was excluded due to correlation with location.
- Brown bears: body weight, latitude, longitude, year, and season, with animal ID included as a random intercept to account for repeated measures. Sex and age were excluded due to correlations with body weight and age class.
- Wolves: body weight, year, latitude, longitude, family group (social unit consisting of breeding pair and offsprings from different years), and age class. Sex and estimated age were excluded due to correlations with body weight.
- Wolverines: sampling year, body weight, and six different locations in Innlandet county, Norway (Evenstadlia, Gravberget, Lia, Opphus, Ottlaugkampen and Veslbyringen). Sex was excluded due to correlation with body weight.

We performed all statistical analyses in R version 4.4.2 [45].

## Results

**Fig. 2:**
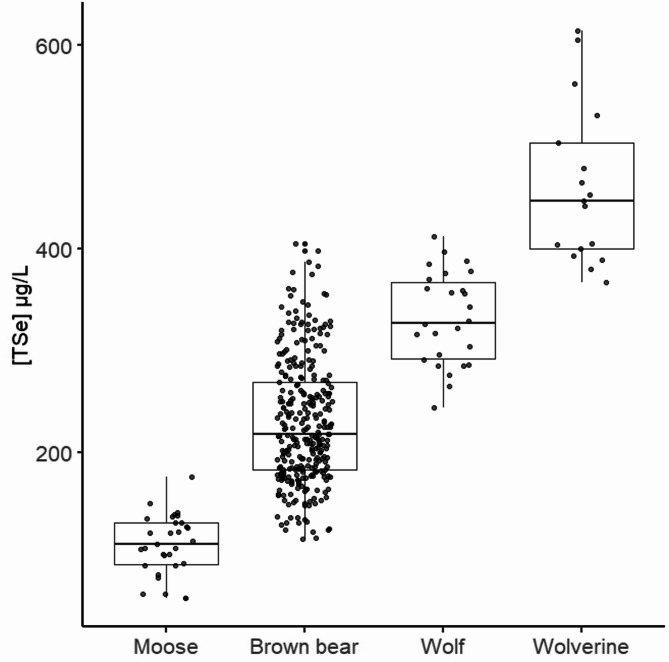
Selenium in a trophic cascade. Significant different concentrations of total selenium ([TSe]) measured in whole blood (µg/L) in four free-ranging Scandinavian wildlife species: Moose (*Alces alces*, *n* = 31), brown bears (*Ursus arctos*, *n* = 305), wolves (*Canis lupus*, *n* = 26) and wolverines (*Gulo gulo*, *n* = 17). Significant differences between all species (*P* < 0.001)

The concentration of the response variable total selenium [TSe] was normally distributed confirmed with histogram and Shapiro Wilkes test. We observed a pattern of a significant difference in [TSe] measured in whole blood between different species (F_3,375_=156.7, *P* < 0.001, Fig. 2), with a post hoc test confirming significant differences between all species (Table 3). Specifically, moose had significantly lower mean values compared to brown bears (estimated difference = 120 µg/L, SE = 11.0 µg/L, t_375_=-10.94, *P* < 0.001) and wolves (estimated difference = 222 µg/L, SE = 15.5 µg/L, t_375_=-14.29, *P* < 0.001). Similarly, brown bears differed significantly from wolverines (estimated difference = 231 µg/L, SE = 14.6 µg/L, t_375_= -15.87, *P* < 0.001). The largest observed difference was between moose and wolverines, with an estimated marginal mean difference of 352 µg/L (SE = 17.6 µg/L, t_375_=-19.94, *P* < 0.001). All pairwise contrasts were significant, with the smallest effect size observed between wolves and wolverines (estimated difference = 130 µg/L, SE = 18.2 µg/L, t_375_=-7.11, *P* < 0.001). Table 3 additionally compares our results with published reference ranges for selected domesticated species. We note that 55% of moose and 5% of brown bears tested in this study have [TSe] below the recommended reference range for selected domesticated species, whereas 49% of bears, 42% wolves and 29% wolverines are displaying [TSe] above the upper reference limit for selected domesticated species. We interpret species-level differences as observed patterns that may reflect both trophic ecology and environmental variation, rather than strictly species-specific physiology.

**Table 3:** Mean ± 2SE and range of total selenium measured in whole blood (µg/L) in four Scandinavian wildlife species

Species	*n*	Mean ± 2SE	Range	Comparable species (reference range)
Moose	31	91.7 ± 8.7	57.0–176.5	Sheep (120–200), Goat (120–350), Cattle (80–120)
Brown bear	305	230.2 ± 6.9	115.0–405.5	Pig (180–220)
Wolf	26	325.9 ± 18.3	244.0–412.0	Dog (220–340)
Wolverine	17	451.5 ± 45.2	367.5–614.0	Mink (142–497)

Moose (*Alces alces*), brown bears (*Ursus arctos*), wolves (*Canis lupus*) and wolverines (*Gulo gulo*). Reference ranges for a selection of comparable domesticated species included [46–48]. Domestic reference ranges were selected to reflect species with comparable diets or physiology, rather than aiming for exhaustive inclusion

### Moose

**Fig. 3:**
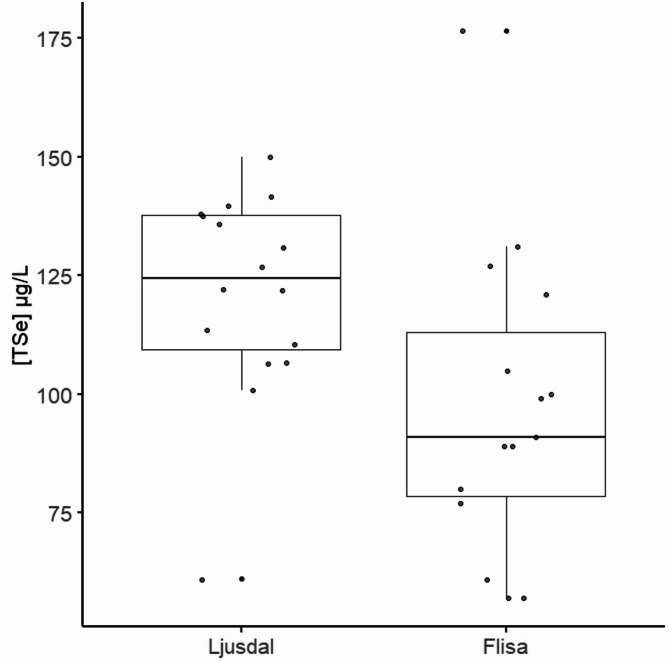
Selenium in moose. Significant different concentrations of total selenium ([TSe]) measured in whole blood (µg/L) in free-ranging Moose (*Alces alces*, *n* = 31) sampled in Ljusdal (Sweden) and Flisa area (Norway)

Model selection using AICc identified the model including both location and sex as the best-fitting model to explain variation in [TSe] in moose (AICc = 286.1, ΔAICc = 0, df = 4). The second-best model included location alone (ΔAICc = 1.4, df = 3). Post hoc comparisons of location, averaged across sex, revealed a significant difference between Ljusdal and Flisa (estimated difference = 24.4 µg/L, SE = 9.43 µg/L, t_27_ = 2.59, *P* = 0.015), where Ljusdal had higher predicted mean [TSe] (125 µg/L, 95% CI: 111.6 µg/L–139.0 µg/L) compared to Flisa (101 µg/L, 95% CI: 85.6 µg/L–116.0 µg/L, Fig. 3). Further, post hoc testing found the difference in [TSe] between sexes estimated at 21 µg/L (SE = 10.6 µg/L, t_27_ = − 1.98, *P* = 0.059), suggesting a trend for higher concentrations in males compared to females.

### Brown bears

**Fig. 4:**
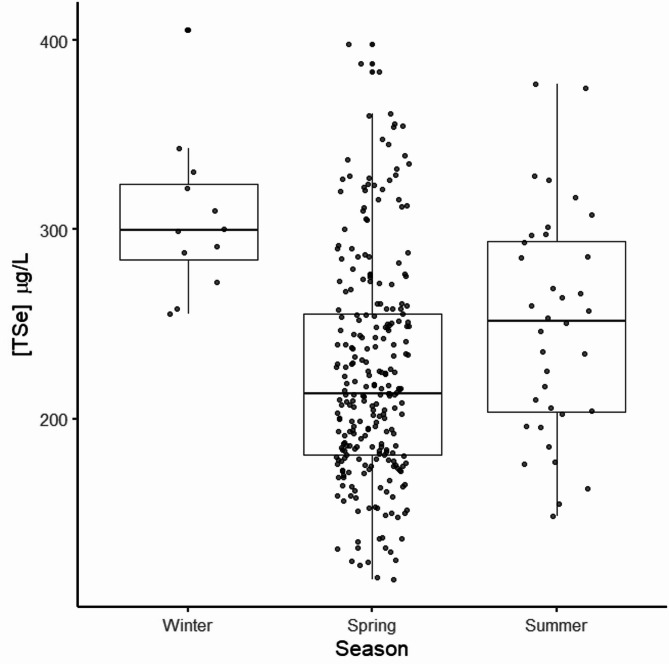
Selenium in brown bears. Significant different concentrations of total selenium (TSe) measured in whole blood (µg/L) sampled from free-ranging brown bears (*Ursus arctos*, *n* = 305) during three different seasons from 2010 to 2021

The best-supported model, with the lowest AICc (3050.5, ΔAICc = 0.00, weight = 0.251), included body weight (Model averaging β = 15.40 µg/L, SE = 3.30 µg/L, z = 4.66, *P* < 0.001)) and season (with winter (β = 95.67, SE = 16.12 µg/L, z = 5.93, *P* < 0.001) and summer (β = 23.83 µg/L, SE = 10.47 µg/L, z = 2.28, *P* = 0.023) compared to spring) as fixed effects and a random intercept for animal ID. The parameter estimates included body weight: 15.43 µg/L (SE = 3.28 µg/L, t = 4.70), indicating that [TSe] increased by approximately 15.43 µg/L for each unit increase in body weight. Additionally, for season (summer): 24.13 (SE = 10.41 µg/L, t = 2.32), suggesting higher selenium concentrations in summer compared to the reference season spring with 219 ± 3.64 µg/L (estimated marginal mean ± SE). The season winter had 95.98 µg/L (SE = 16.05 µg/L, t = 5.98), indicating higher selenium concentrations in winter compared to spring (Fig. 4). The variance attributed to animal ID (random intercept) was estimated at 149.2 µg/L (SD = 12.22 µg/L), and the residual variance was 2761.6 µg/L (SD = 52.55 µg/L). The second-best model included body weight, season, and year as fixed effects, with a marginally higher AICc (3050.6, ΔAICc = 0.15, weight = 0.232). Models with ΔAICc < 2 are presented in Table 4.

**Table 4:** Model selection table for brown bears. Here we present all models with ΔAICc < 2 for [TSe] measured in µg/L whole blood in brown bears

Intercept	Body weight	Year	Lattitude	Longitude	Season	df	AICc	ΔAICc	Model Weight
219.3	15.43				+	6	3050.5	0.00	0.251
-3407.0	15.60	1.80			+	7	3050.6	0.15	0.232
219.2	15.07			-3.196	+	7	3051.7	1.20	0.137
-3157.0	15.29	1.67		-2.671	+	8	3052.1	1.65	0.110

### Wolves

Model selection using AICc identified the model including age class and family group (AICc = 269.2, ΔAICc = 0, weight = 0.152) as the best fit. Posthoc testing revealed significantly higher [TSe] in adult animals compared to yearlings 79.57 µg/L (SE = 24.9 µg/L, t_11_ = − 3.20, *P* = 0.021), whereas no significant difference could be found between family groups. The second-ranked model (ΔAICc = 0.25, weight = 0.134) additionally included longitude, though its inclusion provided only marginal difference in model performance. Other models with ΔAICc < 2 incorporated body weight or year sampled (Table 5).

**Table 5:** Model selection table for wolves. Here we present all models with ΔAICc < 2 for [TSe] measured in µg/L whole blood in wolves

Intercept	Body weight	Age class	Year	Family group	Latitude	Longitude	df	AICc	ΔAICc	Weight
252.60		30.95		4.18			4	269.2	0.00	0.152
135.50		30.76		4.61		8.883	5	269.4	0.25	0.134
-8751.00		37.35	4.45	4.25			5	269.7	0.53	0.117
294		25.88					3	270.9	1.72	0.064

### Wolverines

**Fig. 5:**
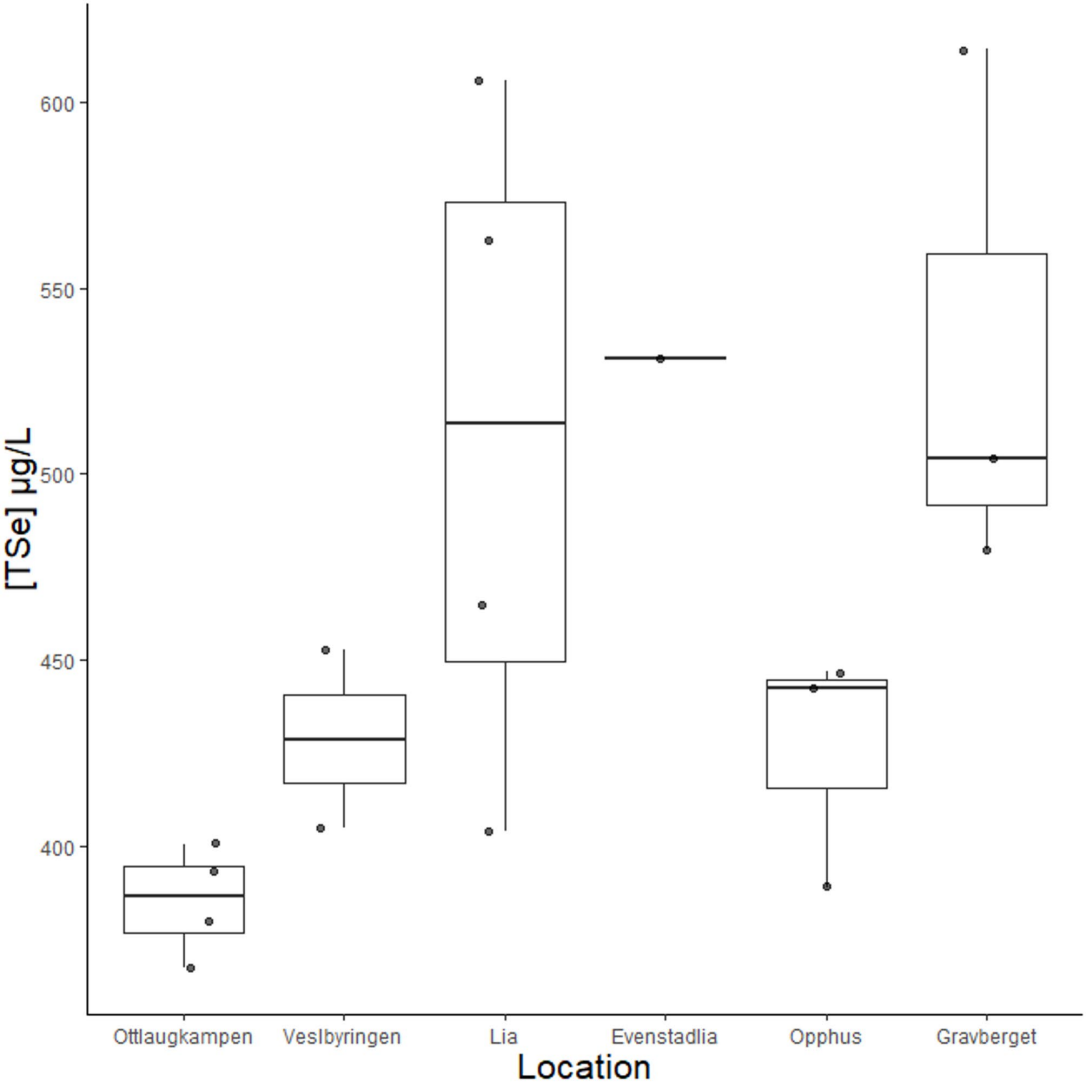
Selenium in wolverines. Total selenium concentration ([TSe]) measured in whole blood in µg/L in wolverines sampled at different locations (organized geographically north – south) in Innlandet county, Norway

Model selection using AICc identified the model that included location only as highest ranked model (AICc = 192.7, ΔAICc = 0, weight = 0.666), Fig. 5). Other models had ΔAICc > 2. Posthoc testing revealed significantly higher Se for animals sampled at Gravberget compared to Ottlaugkampen (estimated difference 147. 24 µg/L, SE = 45.6 µg/L, t_11_ = 3.227, *P* = 0.067).

## Discussion

In this study we documented whole blood selenium concentrations ([TSe]) in four Scandinavian wildlife species with varying trophic positions and found concentrations comparable with selenium deficiency in selected domesticated species (Table 3) in more than half of the moose and in 5% of the brown bears sampled. Surprisingly, 49% of brown bears, 42% wolves and 29% wolverines had [TSe] above recommended blood reference ranges for selected domesticated species, thus not reflecting the poor Se ecosystem. Additionally, we found that [TSe] were significantly associated with location of the animal (all species), body weight/sex/age of the animal (moose, brown bears and wolves all had at least one of these correlated variables included in the best fitted model). Year and season also affected [TSe] with annual variations in brown bears and wolves, and seasonal variations in brown bears. The blood [TSe] increased along the trophic levels: herbivorous moose < omnivorous bears < carnivorous wolves < scavenging wolverines, suggesting biomagnification from deficient to excessive concentrations in large Scandinavian mammals.

Soil Se concentrations in the interior regions of south-central Scandinavia are low, limiting the available [TSe] in the diet of these animals [22]. Our results show that especially the herbivorous moose are affected by this, but also 5% of the omnivorous brown bears had blood [TSe] considered to represent deficiency in domesticated omnivorous pigs. A previous study in Sweden found 50–60% of moose livers with [TSe] that would be considered deficient in cattle [24]. To our knowledge, this is the first Se survey of moose in Scandinavia measuring whole blood, and additionally the first survey of Se status in both Norway and Sweden for the other wildlife species described in this study. Due to the Se poor ecosystem, we were surprised that animals in the higher trophic positions did not reflect these low concentrations since they are foraging on moose with relatively low [TSe], rather they displayed levels above reference ranges for comparable domesticated species. It should be emphasized that we do not have established reference ranges for any of these free-ranging species, and possibly, they are within the normal range for their respective species. Selenosis most commonly occurs from overdosing Se supplements but can be endemic in certain regions of the world where seleniferous soil leads to concentrated Se plants and forage [49]. Typical symptoms of acute toxicity occur with Se whole blood concentrations between 2.2 and 20 ppm across species, which is 3.5 times higher than the highest concentration found in this study (0.614 ppm/614 µg/L). Acute toxicity can include cardiopulmonary lesions, while chronic toxicity can include emaciation, lameness, immunotoxicity, anemia, cardiotoxicity, and poor reproductive performance [50]. No apparent selenosis symptoms were detected in our wildlife species, other than the relatively high whole blood concentrations. We can only speculate why these animals had these high concentrations of Se, despite living in a Se poor ecosystem. One explanation, that these apex predators are exposed to Se supplements from livestock (i.e. mineral bolus in rumen), can likely be dismissed due to the spatial segregation of livestock and carnivores particularly the sampled wolves (The Norwegian zonal carnivore management system [51]). The wolverines especially stand out with high Se concentrations in this study, with significantly higher concentrations than wolves. This may reflect that wolf primarily prey on moose, whereas wolverines as scavengers also prey on species with higher trophic positions. Wolverines’ diet can periodically be dominated by bones and skin [30] which may contain higher [TSe].

Se availability in plants is related to the Se in soil, which again relies on Se composition of the bedrock [19, 22], and this probably contributed to the relatively high variability between local regions for all species sampled in this study. Since moose were sampled in different locations in different years (2018 and 2019 in Flisa and 2020 in Ljusdal) we cannot exclude that the year the moose was tested contributed to the spatial differences. Regional differences were also detected in the Swedish moose survey [24], and in studies investigating Se concentrations in livestock and plants [22]. The annual variation detected for brown bears and wolves is most likely related to annual fluctuation in precipitation [52, 53]. Different seasons will also produce different amounts of rainfall, and previous studies have detected a seasonality in Se geochemistry [54]. A study from Poland found highest Se concentrations during spring [55] in Se deficient roe deer (*Capreolus capreolus*). We found a seasonal difference in brown bears, which were the only species we tested during different seasons. The bears had significantly higher Se during winter, followed by a decrease into spring, and higher concentrations again in the summer. Due to bears’ hibernating nature (winter samples collected during hibernation [25], we cannot exclude that the varying Se concentrations are related to internal physiological changes in the brown bear, rather than Se accessibility. Especially since the Se concentration for brown bears was significantly higher during winter, when they are hibernating and not eating. The other species sampled were all sampled in winter and may have seasonal differences as well.

In all species except for wolverines, we found significant associations with physiological variables such as sex (moose), body weight (bears) or age class (wolves). Body weight, sex and age are highly correlated in these species, and it seems likely that selenium increases with age and/or body weight. This is not surprising and is a well-established fact in domesticated animals and humans, where different age classes are separated for reference ranges [56].

Despite using a unique dataset that includes several free-ranging mammalian species, our study has important limitations. The samples were drawn from existing biobanks associated with long-term research projects in Norway and Sweden, and not all species were sampled with equal intensity or geographic coverage. Bear samples (*n* = 305) were collected over more than a decade and comprise the majority of the dataset. In contrast, we had smaller sample sizes for moose (*n* = 31), wolves (*n* = 26), and wolverines (*n* = 17), collected over shorter periods and in more spatially limited areas.

Although there is partial geographic overlap between species, uneven spatial distribution and sample sizes introduce the possibility of spatial confounding, which complicates efforts to isolate species effects from regional variation in selenium availability. To mitigate this, we included spatial covariates (e.g., latitude and longitude or region) in our models, and we interpret species-level differences as observed patterns that may reflect both trophic ecology and environmental variation, rather than strictly species-specific physiology.

In particular, wolverines showed relatively high [TSe] despite limited sample size and restricted spatial distribution, and we suggest comparing regions where sheep are known to be killed by wolverines, to regions without livestock mortalities, to explore if supplemented livestock are driving the relatively high [TSe]. Additionally, repeated measurements of [TSe] in brown bears sampled multiple times present an interesting opportunity for future research. Such an analysis could help elucidate how selenium concentrations develop or change over time within individuals, offering insights into long-term selenium dynamics in this species.

The relatively low [TSe] documented in moose and bears in this study has been observed in domesticated livestock species in Scandinavia for decades [21], and livestock often receive Se supplements [56]. Finland is the only Scandinavian country that adds supplemental Se (sodium selenate) to fertilizers nationwide, bringing the country from widely deficient levels in the 1970s to significantly increased blood concentrations of Se in both animals and humans [57]. Full understanding of the health benefits in Finland from this intervention will require a multidisciplinary approach, but increased cancer risk in humans has been linked to Se deficient diets [58]. Se protects against oxidative stress as a potent antioxidant and as a vital part of glutathione peroxidase [4, 56]. The biochemical properties of Se, where it can bind to and in-activate heavy metals [5, 7, 17, 59] is an additional health benefit from Se. Communities with high fish and/or marine mammal consumption should be aware of these benefits, and maybe other Nordic countries could benefit from following Finland’s example and add Se to fertilizers (Fig. 5).

## Conclusions

In summary, this study found strong relationships between different wildlife megafauna’s concentration of Se in whole blood with location of the animal, age class/body weight or sex of the animal, and annual or seasonal changes. More than half of moose sampled had relatively low Se concentrations, as well as 5% of the brown bears. Surprisingly, apex predators expressed relatively high Se concentrations. The relatively low Se concentrations found in moose and some bears should be accounted for in ecotoxicology studies, since the documented Se deficiency in Scandinavia can exacerbate heavy metal toxicosis.

## Data Availability

The datasets used and/or analyzed during the current study are available from the corresponding author on reasonable request.
